# Dynamic Quantitative Trait Loci Analysis of Seed Reserve Utilization during Three Germination Stages in Rice

**DOI:** 10.1371/journal.pone.0080002

**Published:** 2013-11-11

**Authors:** Xinxin Cheng, Jinping Cheng, Xi Huang, Yanyan Lai, Ling Wang, Wenli Du, Zhoufei Wang, Hongsheng Zhang

**Affiliations:** The Laboratory of Seed Science and Technology, State Key Laboratory of Crop Genetics and Germplasm Enhancement, Nanjing Agricultural University, Nanjing, PR China; Institute of Genetics and Developmental Biology, Chinese Academy of Sciences, China

## Abstract

In this study, one rice population of recombinant inbred lines (RILs) was used to determine the genetic characteristics of seed reserve utilization during the early (day 6), middle (day 10) and late (day 14) germination stages. The seedling dry weight (SDW) and weight of the mobilized seed reserve (WMSR) were increased, while the seed reserve utilization efficiency (SRUE) decreased, during the process of seed germination. The SDW and WMSR were affected by the seed weight, while the SRUE was not affected by the seed weight. A total of twenty unconditional and twenty-one conditional additive QTLs and eight epistatic QTLs were identified at three germination stages, and the more QTLs were expressed at the late germination stage. Among them, twelve additive and three epistatic QTLs for SDW, eight additive and three epistatic QTLs for WMSR and thirteen additive and two epistatic QTLs for SRUE were identified, respectively. The phenotypic variation explained by each additive QTL, epistatic QTL and QTL × development interaction ranged from 6.10 to 23.91%, 1.79 to 6.88% and 0.22 to 2.86%, respectively. Two major additive QTLs *qWMSR7.1* and *qSRUE4.3* were identified, and each QTL could explain more than 20% of the total phenotypic variance. By comparing the chromosomal positions of these additive QTLs with those previously identified, eleven QTLs might represent novel genes. The best four cross combinations of each trait for the development of RIL populations were selected. The selected RILs and the identified QTLs might be applicable to improve rice seed reserve utilization by the marker-assisted selection approach.

## Introduction

Vigorous seedling growth is essential for profitable and sustainable crop production. Seedling establishment begins with the appearance of the radicle and terminates when the seedling has exhausted the seed's energy reserves and starts to carry out photosynthesis [1]. Germination and heterotrophic growth are therefore crucial steps for the establishment of crops. By definition, seed vigor is the ability of a seed lot to establish seedlings successfully under a wide range of conditions [2]. High seed vigor, i.e., the rapid, uniform and complete emergence of vigorous seedlings, leads to high competition against weeds, uniform harvest and a high grain yield potential in rice. Improving rice seed vigor is an important objective in rice breeding programs.

Seed vigor is a complex trait that is determined by the interactions between genetic and environmental factors [3]. To date, several technological solutions, such as seed selection and seed treatment, have been used to improve seed vigor [2], [4]–[7]. In recent years, the emphasis on improving seed vigor has shifted to the potential of genetic improvement in breeding programs. Quantitative trait loci (QTL) analysis for seed vigor had been reported in rice, tomato and lettuce [8]–[13]. Those QTLs can be used to improve seed vigor using a marker-assisted selection (MAS) breeding program; furthermore, it becomes possible to identify candidate genes associated with seed vigor [14].

As seeds imbibe water, the mobilization of seed reserves such as starch, proteins, and lipids from storage tissues or the endosperm begins, providing essential energy to fuel germination and seedling growth until the seedling becomes photoautotrophic [15]. Heterotrophic seedling growth (seedling dry weight; SDW) can be defined as a product of two key traits: the weight of the mobilized seed reserve (WMSR) and the conversion efficiency of the utilized seed reserve (seed reserve utilization efficiency; SRUE) into seedling tissue [16]–[17]. These traits of seed reserve utilization play important roles in high seed vigor to improve germination and heterotrophic growth. Several phenotypic studies of seed reserve utilization have been conducted in wheat [16], soybean [17] and chickpea [18], but no studies have been reported in rice. Furthermore, the genetic control of seed reserve utilization in rice remains uncharacterized.

In rice, QTLs for seed vigor traits such as seed germination, seedling growth, seed longevity and tolerance to stress have been identified [8], [11]–[13], [19]–[25]. In most of these reports, the phenotypic values of the final developmental stage were used for QTL analysis. These QTLs with unconditional genetic effects (defined by Sen and Churchill [26]) are effective throughout seed germination. According to the theory of developmental genetics, genes are expressed selectively at different germination stages. QTL analysis can be adapted to include the effects of developmental stages [27]. Therefore, the QTL with conditional genetic effects (defined by Sen and Churchill [26]) are the loci detected at a specific growth stage [28]. The distinct gene actions for seed vigor at different developmental stages have been ignored in previous studies. Identification of both conditional and unconditional QTL will be desirable for MAS [28]. It will be essential, therefore, to include the dynamics of gene expression when analyzing the traits of seed reserve utilization.

Recently, several reports have explored the developmental behavior of various quantitative traits in rice [29]–[30], maize [31], cotton [32], soybean [28] and potato [33]. In rice, the dynamic QTL analysis has been used to dissect the genetic control of several quantitative traits such as tiller number [29]–[30], grain-filling [34] and root morphological traits [35]. The objectives of the present study were to investigate the developmental behavior of seed reserve utilization in rice, to identify both conditional and unconditional QTL and to measure the epistasis effects on seed reserve utilization during seed germination.

## Materials and Methods

### Plant materials

Two rice varieties, Jiucaiqing (*japonica*) and IR26 (*indica*), and 150 recombinant inbred lines (RIL) (F_2:9_) derived from Jiucaiqing × IR26 were used in this study [19]–[20]. All materials were grown at the Jiangpu Experimental Station of Nanjing Agricultural University, and the seeds were harvested at the mature stage. After the release of seed dormancy by post-ripening after four months of storage at room temperature (22–26°C), the seeds were used for the germination test.

### Evaluation of seed reserve utilization

To determine the seed water content (WC) of each RIL and their parents, three replicates of seed (∼4.0 g per replicate) were powdered and dried at 104°C for 24 h. For the evaluation of seed reserve utilization, three replicates of each RIL and their parents (thirty seeds per replicate) were used and weighed (W). According to the corresponding WC, the initial seed dry weight (W0) (ISDW; mg per seed) of each RIL and their parents were calculated as [W×(1−WC)/30].

Then, the seed germination was conducted in a growth chamber at 30±1°C in the dark for 6, 10 or 14 days according to the methods of Zhang et al. [24]. The shoots and roots of seedlings were separated from the seeds by hand after germination. The seedlings dry weight (W1) (SDW; mg per seed) and the remnant seed dry weight (W2) (RSDW; mg per seed) were obtained after oven drying at 104°C for 24 h. The weight of the mobilized seed reserve (W3) (WMSR; mg per seed) was calculated as [W0–W2]. The seed reserve utilization efficiency (SRUE; mg per mg) was calculated as [W1/W3] [16].

### Data analysis

Experimental data were analyzed using the Statistical Analysis System (SAS) software, and the traits of the parents were compared using Student's *t*-test at the 5% and 1% levels of probability. The correlations of the traits were computed using the PROC CORR by SAS software [19]–[20].

### QTL mapping

The linkage map has been constructed previously [19]–[20]. Finally, a set of 168 SSR markers covering most of the rice genome at an average interval of 15 cM was constructed. Unconditional QTL was detected using the mean phenotypic values at day 6, 10 and 14 of germination. The genetic behavior measured at time *t* is the result of genes expressed before time (*t*-1) and extra effects within the period from (*t*-1) to *t*. Conditional phenotypic values (*t/t-1*) at time *t* were given by subtracting the phenotypic means measured at time *t*-1 from the mean at time *t* [27], [36]. Conditional QTL was detected using the mean phenotypic values (*t/t-1*) in the intervals from day 0 to 6 (*t_6_/t_0_*), 6 to 10 (*t_10_/t_6_*) and 10 to 14 (*t_14_/t_10_*) of germination.

Using the phenotypic values at a single germination stage, the unconditional and conditional additive QTL was identified respectively by the inclusive composite interval mapping (ICIM) method in QTL IciMapping [37]–[38]. Briefly, the P values for entering variables (PIN) and removing variables (POUT) were set at 0.01 and 0.02, and the scanning step was 1 cM [19]. The LOD score of 2.5 was used as the threshold value to declare the presence of a putative additive QTL. The QTLNetwork program ver. 2.0, based on a mixed linear model [39] was used to identify the epistatic QTL for seed reserve utilization in joint analysis of the unconditional phenotypic values of three germination stages. Briefly, the values for testing window and filtration window were set at 10 cM, respectively, and the walking speed was 1 cM. The putative QTL detection was determined at 95% confidence level. The proportion of observed phenotypic variance explained by each additive and epistatic QTL and the corresponding additive effects were also estimated. The QTL nomenclature followed the method of McCouch and CGSNL [40].

### Prediction for novel parental combination

The best cultivars with maximum phenotypic value and elite alleles might be used to design parental combinations for crop breeding [41]. According to the information of phenotypic values and alleles of detected loci among the RILs, the best four cross combinations of each trait were selected. Firstly, the RILs with relatively highest phenotypic values of SDW, WMSR or SRUE at three germination stages (Day 6, 10 and 14) were selected. Then, the positive alleles of the detected additive QTLs among the selected RILs were analyzed. Using Monte Carlo simulation experiments, a novel cultivar combination can be determined by maximizing the number of elite alleles [41]. Finally, the best four cross combinations of each trait were selected.

## Results

### Seed reserve utilization phenotypes

During the process of seed germination, the SDW and WMSR were increased among the parents and RILs, while the SRUE was decreased. There were significant differences in the SDW, WMSR and SRUE between two parents at the middle (Day 10) and late (Day 14) germination stages (Table 1). The SDW and WMSR of Jiucaiqing were significantly higher than those of IR26 at the middle and late germination stages. In contrast, IR26 had a significantly higher SRUE at the middle and late germination stages. There was a continuous frequency distribution and transgressive segregation in the SDW, WMSR and SRUE among RIL population at three germination stages.

**Table 1:** Phenotypic values of seed reserve utilization related traits among the parents and RILs population at three germination stages.

Germination stages (d)	Traits^a^	Parents^b^		RIL Population^c^					
		Jiucaiqing	IR26	Mean	Max	Min	SD	Skewness	Kurtosis
6	SDW	2.81±0.14	2.54±0.37	3.73	5.96	1.41	0.88	−0.06	−0.04
	WMSR	3.50±0.49	3.33±0.26	4.33	8.25	1.74	1.21	0.48	0.28
	SRUE	0.80±0.17	0.76±0.09	0.86	0.99	0.33	0.11	−1.57	3.78
10	SDW	4.55±1.02**	3.71±0.81	5.74	9.77	2.61	1.29	0.68	1.03
	WMSR	10.52±0.27**	4.88±0.60	8.85	15.95	4.40	1.90	0.93	1.90
	SRUE	0.43±0.04	0.76±0.02**	0.65	0.80	0.48	0.06	−0.16	0.04
14	SDW	6.29±0.85**	5.43±1.49	6.93	11.13	2.53	1.32	0.12	0.57
	WMSR	11.44±0.48**	8.43±1.15	10.79	16.28	5.09	1.84	0.13	0.40
	SRUE	0.55±0.03	0.64±0.13**	0.64	0.94	0.32	0.07	−0.58	5.63

a SDW: seedling dry weight, mg per seed; WMSR: weight of mobilized seed reserve, mg per seed; SRUE: seed reserve utilization efficiency, mg per mg;

b Means ± SD (standard deviation);

**indicates significance at the level of 1% according to Student's *t*-test;

c RILs sample size n = 150, replications r = 3.

### Correlation of seed weight with seed reserve utilization related traits

There were significant positive correlations between the ISDW and SDW during three germination stages (Table 2). The correlations between the ISDW and the WMSR were significantly positive during the middle and late germination stages, but there were no significant correlations between ISDW and SRUE. These results indicated that the SDW and WMSR are affected by the seed dry weight, while not the SRUE.

**Table 2:** Correlation coefficients between seed reserve utilization related traits and seed weight at three germination stages.

Traits^a^ (Day 6)	ISDW		Traits (Day 10)	ISDW		Traits (Day 14)	ISDW	
	*t* b	*t/t-1* c		*t* b	*t/t-1* c		*t* b	*t/t-1* c
SDW	0.312**	0.312**	SDW	0.601**	0.379**	SDW	0.683**	0.259**
WMSR	−0.187	−0.187	WMSR	0.606**	0.488**	WMSR	0.736**	0.249**
SRUE	0.073	0.073	SRUE	0.113	0.051	SRUE	0.086	−0.089

a ISDW: initial seed dry weight; SDW: seedling dry weight; WMSR: weight of mobilized seed reserve; SRUE: seed reserve utilization efficiency;

b Unconditional phenotypic values at the day 6, 10 and 14 of germination;

c Conditional phenotypic values in the intervals from day 0 to 6 (*t_6_/t_0_*), 6 to 10 (*t_10_/t_6_*) and 10 to 14 (*t_14_/t_10_*) of germination;

**indicates significance at the level of 1%.

### Unconditional and conditional QTL for SDW

**Figure 1:**
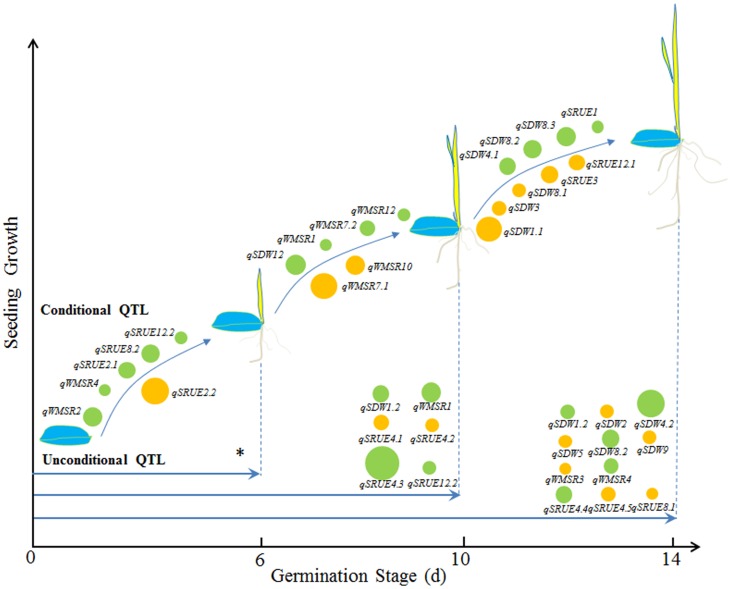
Unconditional and conditional QTL for seed reserve utilization related traits at three germination stages. * The unconditional QTL at day 6 stage is same with the conditional QTL during day 0 to 6 stage. The circle size represents the magnitude of phenotypic variance explained by each QTL. Yellow circle indicates that the positive allele of Jiucaiqing could increase the value of traits. Green circle indicates that the positive allele of IR26 could increase the value of traits.

A total of six unconditional additive QTLs were identified for the SDW at three germination stages (Table 3; Fig. 1). One QTL (*qSDW1.2*) and six QTLs (*qSDW1.2*, *qSDW2*, *qSDW4.2*, *qSDW5*, *qSDW8.2* and *qSDW9*) were detected at day 10 and 14 stage, respectively. Among them, the *qSDW1.2* was consistently detected in both germination stages. The phenotypic variance explained by each QTL ranged from 6.78 to 16.75%. The positive alleles of three QTLs (*qSDW2*, *qSDW5* and *qSDW9*) from Jiucaiqing and three QTLs (*qSDW1.2*, *qSDW4.2* and *qSDW8.2*) from IR26 contributed to the increase of SDW.

**Table 3:** Unconditional and conditional QTLs for seed reserve utilization related traits at three germination stages.

Traits^a^	Germination stage (d)	*t* b						*t/t-1* c					
		QTLs	Chr.^d^	Marker interval	LOD	r^2^ (%)^e^	A^f^	QTLs	Chr.^d^	Marker interval	LOD	r^2^ (%)^e^	A^f^
SDW	10	*qSDW1.2*	1	RM6950–RM5759	3.96	12.93	−0.54	*qSDW12*	12	RM519–RM7376	4.99	15.63	−0.50
	14	*qSDW1.2*	1	RM6950–RM5759	3.41	10.92	−0.51	*qSDW1.1*	1	RM8105–RM259	6.38	16.18	0.43
		*qSDW2*	2	RM5651–RM5804	3.16	6.78	0.35	*qSDW3*	3	RM3684–RM6712	3.45	8.51	0.30
		*qSDW4.2*	4	RM273–RM3839	7.06	16.75	−0.54	*qSDW4.1*	4	RM16626–RM5687	4.14	9.04	−0.30
		*qSDW5*	5	RM159–RM7302	2.97	8.29	0.38	*qSDW8.1*	8	RM72–RM7027	3.11	6.97	0.30
		*qSDW8.2*	8	RM342–RM3459	4.43	14.36	−0.54	*qSDW8.2*	8	RM342–RM3459	2.73	9.95	−0.34
		*qSDW9*	9	RM6570–RM3164	3.07	7.09	0.36	*qSDW8.3*	8	RM7356–RM7556	4.80	10.82	−0.39
WMSR	6	*qWMSR2*	2	RM240–RM5404	4.82	13.44	−0.48	*qWMSR2*	2	RM240–RM5404	4.82	13.44	−0.48
		*qWMSR4*	4	RM3839–RM3367	2.67	8.40	−0.36	*qWMSR4*	4	RM3839–RM3367	2.67	8.40	−0.36
	10	*qWMSR1*	1	RM6950–RM5759	4.80	14.60	−0.84	*qWMSR1*	1	RM6950–RM5759	2.80	9.83	−0.68
								*qWMSR7.1*	7	RM6872–RM11	6.79	20.51	0.83
								*qWMSR7.2*	7	RM5380–RM7564	3.69	11.03	−0.61
								*qWMSR10*	10	RM3451–RM228	4.76	12.16	0.64
								*qWMSR12*	12	RM519–RM7376	2.93	8.04	−0.53
	14	*qWMSR3*	3	RM282–RM6080	2.57	9.04	0.57						
		*qWMSR4*	4	RM3839–RM3367	3.60	10.20	−0.59						
SRUE	6	*qSRUE2.1*	2	RM5631–RM3685	4.03	11.02	−0.04	*qSRUE2.1*	2	RM5631–RM3685	4.04	11.02	−0.04
		*qSRUE2.2*	2	RM240–RM5404	7.12	18.65	0.05	*qSRUE2.2*	2	RM240–RM5404	7.12	18.65	0.05
		*qSRUE8.2*	8	RM6976–RM6845	4.50	10.15	−0.04	*qSRUE8.2*	8	RM6976–RM6845	4.50	10.15	−0.04
		*qSRUE12.2*	12	RM5609–RM6953	3.01	8.22	−0.03	*qSRUE12.2*	12	RM5609–RM6953	3.01	8.22	−0.03
	10	*qSRUE4.1*	4	RM335–RM518	3.66	9.54	0.02						
		*qSRUE4.2*	4	RM518–RM16535	3.22	7.67	0.02						
		*qSRUE4.3*	4	RM16626–RM5687	8.91	23.91	−0.03						
		*qSRUE12.2*	12	RM5609–RM6953	3.15	10.79	−0.01						
	14	*qSRUE4.4*	4	RM7187–RM3687	4.15	14.59	−0.03	*qSRUE1*	1	RM5536–RM3362	2.77	6.10	−0.07
		*qSRUE4.5*	4	RM3306–RM8220	3.36	9.22	0.02	*qSRUE3*	3	RM6832–RM135	4.15	12.37	0.11
		*qSRUE8.1*	8	RM7027–RM6215	2.58	6.69	0.02	*qSRUE12.1*	12	RM20–RM6296	3.80	10.86	0.11

a SDW: seedling dry weight; WMSR: weight of mobilized seed reserve; SRUE: seed reserve utilization efficiency;

b Unconditional QTL at the day 6, 10 and 14 of germination;

c Conditional QTL in the intervals from day 0 to 6 (*t_6_/t_0_*), 6 to 10 (*t_10_/t_6_*) and 10 to 14 (*t_14_/t_10_*) of germination;

d Chromosome on which the QTL was located;

e Variation explained by each putative QTL;

f Additive effect is the effect of substituting a Jiucaiqing allele for an IR26 allele; Its positive value indicates that Jiucaiqing has the positive allele; The case of negative values is just the opposite.

Seven conditional additive QTLs were identified for the SDW during three germination stages (Table 3; Fig. 1). One QTL (*qSDW12*) and six QTLs (*qSDW1.1*, *qSDW3*, *qSDW4.1*, *qSDW8.1*, *qSDW8.2* and *qSDW8.3*) were detected during day 6 to 10 stage and day 10 to 14 stage, respectively. The phenotypic variance explained by each QTL ranged from 6.97 to 16.18%. The positive alleles of three QTLs (*qSDW1.1*, *qSDW3* and *qSDW8.1*) from Jiucaiqing and four QTLs (*qSDW4.1*, *qSDW8.2*, *qSDW8.3* and *qSDW12*) from IR26 contributed to the increase of SDW.

### Unconditional and conditional QTL for WMSR

Four unconditional additive QTLs were identified for the WMSR at three germination stages (Table 3; Fig. 1). Two QTLs (*qWMSR2* and *qWMSR4*), one QTL (*qWMSR1*) and two QTLs (*qWMSR3* and *qWMSR4*) were detected at day 6, 10 and 14 stage, respectively. Among them, the *qWMSR4* was consistently detected in both day 6 and 14 stages. The phenotypic variance explained by each QTL ranged from 8.40 to 14.60%. The positive alleles of one QTL (*qWMSR3*) from Jiucaiqing and three QTLs (*qWMSR1*, *qWMSR2* and *qWMSR4*) from IR26 contributed to the increase of WMSR.

Seven conditional additive QTLs were identified for the WMSR during three germination stages (Table 3; Fig. 1). Two QTLs (*qWMSR2* and *qWMSR4*) and five QTLs (*qWMSR1*, *qWMSR7.1*, *qWMSR7.2*, *qWMSR10* and *qWMSR12*) were detected during day 0 to 6 stage and day 6 to 10 stage, respectively. The phenotypic variance explained by each QTL ranged from 8.04 to 20.51%; the major QTL *qWMSR7.1* accounted for the largest amount of phenotypic variation (20.51%) during the day 6 to 10 stage. The positive alleles of two QTLs (*qWMSR7.1* and *qWMSR10*) from Jiucaiqing and five QTLs (*qWMSR1*, *qWMSR2*, *qWMSR4*, *qWMSR7.2* and *qWMSR12*) from IR26 contributed to the increase of WMSR.

### Unconditional and conditional QTL for SRUE

Ten unconditional additive QTLs were identified for the SRUE at three different germination stages (Table 3; Fig. 1). Four QTLs (*qSRUE2.1*, *qSRUE2.2*, *qSRUE8.2*, and *qSRUE12.2*), four QTLs (*qSRUE4.1*, *qSRUE4.2*, *qSRUE4.3* and *qSRUE12.2*) and three QTLs (*qSRUE4.4*, *qSRUE4.5* and *qSRUE8.1*) were detected at day 6, 10 and 14 stage, respectively. Among them, the *qSRUE12.2* was consistently detected in both day 6 and 10 stages. The phenotypic variance explained by each QTL ranged from 6.69 to 23.91%; the major QTL *qSRUE4.3* accounted for the largest amount of phenotypic variation (23.91%) at the day 10 stage. The positive alleles of five QTLs (*qSRUE2.2*, *qSRUE4.1*, *qSRUE4.2*, *qSRUE4.5* and *qSRUE8.1*) from Jiucaiqing and five QTLs (*qSRUE2.1*, *qSRUE4.3*, *qSRUE4.4*, *qSRUE8.2* and *qSRUE12.2*) from IR26 contributed to the increase of SRUE.

Seven conditional additive QTLs were identified for the SRUE during three germination stages (Table 3; Fig. 1). Four QTLs (*qSRUE2.1*, *qSRUE2.2*, *qSRUE8.2* and *qSRUE12.2*) and three QTLs (*qSRUE1*, *qSRUE3* and *qSRUE12.1*) were detected during day 0 to 6 stage and day 10 to 14 stage, respectively. The phenotypic variance explained by each QTL ranged from 6.10 to 18.65%. The positive alleles of three QTLs (*qSRUE2.2*, *qSRUE3* and *qSRUE12.1*) from Jiucaiqing and four QTLs (*qSRUE1*, *qSRUE2.1*, *qSRUE8.2* and *qSRUE12.2*) from IR26 contributed to the increase of SRUE.

### Epistatic QTL for seed reserve utilization

**Figure 2:**
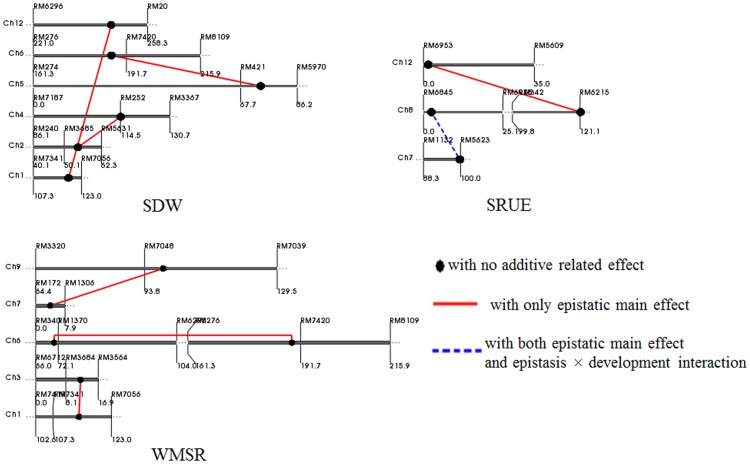
Location of epistatic QTLs for seed reserve utilization related traits on linkage groups in joint analysis of three germination stages. SDW: seedling dry weight; WMSR: weight of mobilized seed reserve; SRUE: seed reserve utilization efficiency. The chromosome names are listed on the top left of the bar; the map distances (cM) are shown below the bar and the markers are shown above the bar.

A total of eight epistatic QTLs were identified by the joint analysis of the unconditional phenotypic values at three germination stages (Day 6, 10 and 14) (Table 4; Fig. 2). Among them, three epistatic QTLs each for SDW and WMSR were identified, respectively, with an epistatic main effect. The SRUE was controlled by two epistatic QTLs: one with both an epistatic main effect and an epistasis × development interaction effect and one with only an epistatic main effect. The phenotypic variance explained by each epistatic QTL ranged from 1.79 to 6.88%, and the phenotypic variation explained by each epistatic QTL × development interaction ranged from 0.22 to 2.86%.

**Table 4:** The epistatic QTL for seed reserve utilization related traits in joint analysis of three germination stages.

Traits^a^	Loci (*i*)^b^		Loci (*j*)^b^		A^c^	AD^c^			r^2^ (A)	r^2^ (/AD)
	Chr.	Intervals	Chr.	Intervals		AD1	AD2	AD3	(%)^d^	(%)^d^
SDW	1	RM7341–RM7056	12	RM6296–RM20	0.58**	−0.12	0.02	0.10	6.16	0.73
	2	RM3685–RM5631	4	RM7187–RM252	0.32**	−0.16	0.03	0.13	4.50	1.18
	5	RM421–RM5970	6	RM276–RM7420	−0.45**	0.01	–0.01	0.01	5.30	0.22
WMSR	1	RM7341–RM7056	3	RM3684–RM3564	0.48**	−0.15	0.12	0.04	5.54	0.93
	6	RM340–RM1370	6	RM276–RM7420	−0.45**	0.12	−0.08	−0.04	4.42	0.71
	7	RM172–RM1306	9	RM7048–RM7039	0.52**	−0.07	0.02	0.05	6.88	0.68
SRUE	7	RM1132–RM5623	8	RM6845–RM6976	0.01**	0.02**	−0.01	−0.01	1.79	2.86
	8	RM342–RM6215	12	RM6953–RM5609	0.02**	0.01	−0.01	−0.01	3.53	1.07

a SDW: seedling dry weight; WMSR: weight of mobilized seed reserve; SRUE: seed reserve utilization efficiency;

b Chromosome on which the QTL was located;

c A represents the estimated additive effect of epistatic QTL, and AD1, AD2 and AD3 represents the additive effects of epistatic QTL at day 6, 10 and 14 of germination, respectively; Its positive value indicates that two loci genotypes being the same as those in parent Jiucaiqing (or IR26) take the positive effects, while the two-loci recombinants take the negative effects;

d r^2^ (A) and r^2^ (AD) represents the phenotypic variation explained by the epistatic QTL and epistatic QTL × development interactions, respectively;

**indicates significance at the level of 1%.

### Prediction for novel parental combination

**Figure 3:**
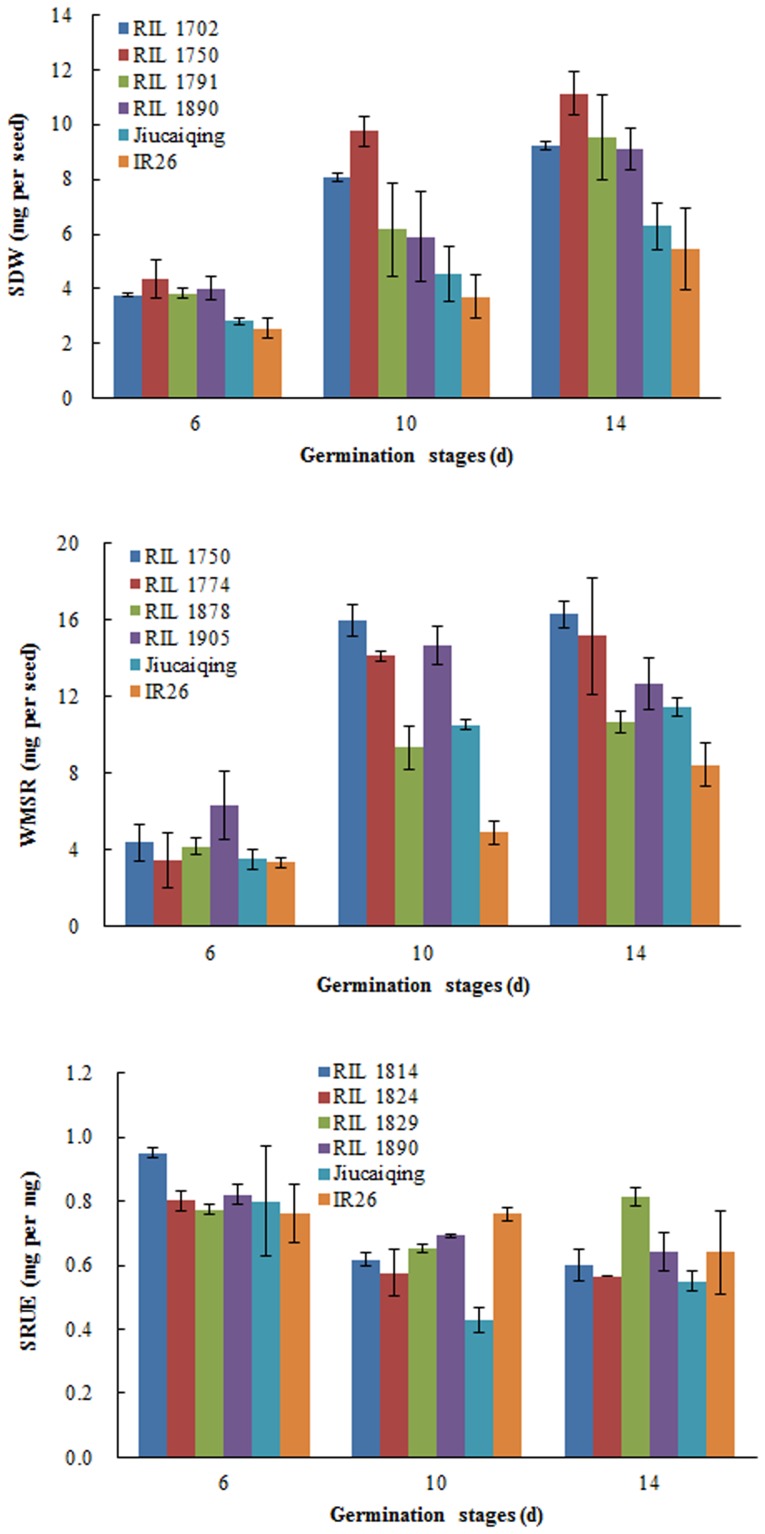
The phenotypes of seed reserve utilization related traits among the selected RILs and two parents at three germination stages.

The best four cross combinations of each trait for the development of RIL populations were selected to improve seed reserve utilization (Table 5). Four RILs each for SDW, WMSR and SRUE were selected with a relatively high value at three germination stages respectively (Fig. 3). The selected RILs had two to eight positive alleles of the additive QTLs for each trait (Table 6). To improve the three traits (SDW, WMSR and SRUE) simultaneously, 33 elite alleles could be pyramided by the combination of RILs 1750, 1791, 1878, and 1890 (Table 6).

**Table 5:** Parental combinations predicted from RILs of seed reserve utilization related traits.

Trait^a^	Parental combination	Trait^a^	Parental combination	Trait^a^	Parental combination
SDW	RIL 1702×RIL 1791	WMSR	RIL 1750×RIL 1774	SRUE	RIL 1814×RIL 1824
	RIL 1702×RIL 1890		RIL 1750×RIL 1878		RIL 1814×RIL 1829
	RIL 1750×RIL 1890		RIL 1774×RIL 1905		RIL 1814×RIL 1890
	RIL 1791×RIL 1890		RIL 1878×RIL 1905		RIL 1829×RIL 1890

a SDW: seedling dry weight; WMSR: weight of mobilized seed reserve; SRUE: seed reserve utilization efficiency.

**Table 6:** The related additive QTLs with positive alleles in selected RILs.

Traits^a^	Detected QTLs	Selected RILs^b^									
		1702	1750	1774	1791	1814	1824	1829	1878	1890	1905
SDW	*qSDW1.1*				*+*					*+*	
	*qSDW1.2*	*+*	*+*	*+*							
	*qSDW2*	*+*		*+*						*+*	
	*qSDW3*	*+*		*+*						*+*	
	*qSDW4.1*	*+*			*+*	*+*		*+*	*+*		
	*qSDW4.2*	*+*		*+*	*+*	*+*	*+*	*+*	*+*		
	*qSDW5*			*+*			*+*	*+*	*+*	*+*	
	*qSDW8.1*									*+*	
	*qSDW8.2*	*+*	*+*		*+*		*+*	*+*	*+*		*+*
	*qSDW8.3*	*+*					*+*	*+*		*+*	*+*
	*qSDW9*								*+*	*+*	
	*qSDW12*	*+*	*+*	*+*					*+*		*+*
WMSR	*qWMSR1*	*+*	*+*	*+*	*+*						
	*qWMSR2*				*+*		*+*	*+*			*+*
	*qWMSR3*									*+*	
	*qWMSR4*		*+*	*+*	*+*	*+*	*+*		*+*		
	*qWMSR7.1*								*+*		
	*qWMSR7.2*		*+*		*+*		*+*	*+*			*+*
	*qWMSR10*	*+*		*+*					*+*	*+*	
	*qWMSR12*		*+*						*+*		
SRUE	*qSRUE1*	*+*	*+*	*+*	*+*	*+*					
	*qSRUE2.1*				*+*			*+*	*+*		
	*qSRUE2.2*					*+*			*+*		
	*qSRUE3*									*+*	
	*qSRUE4.1*						*+*			*+*	*+*
	*qSRUE4.2*						*+*			*+*	*+*
	*qSRUE4.3*	*+*	*+*		*+*	*+*		*+*	*+*		
	*qSRUE4.4*	*+*		*+*			*+*	*+*		*+*	
	*qSRUE4.5*						*+*	*+*		*+*	
	*qSRUE8.1*				*+*	*+*				*+*	
	*qSRUE8.2*	*+*		*+*	*+*	*+*		*+*			
	*qSRUE12.1*							*+*		*+*	
	*qSRUE12.2*	*+*		*+*			*+*		*+*		*+*

a SDW: seedling dry weight; WMSR: weight of mobilized seed reserve; SRUE: seed reserve utilization efficiency;

b + represents the additive QTL with positive allele in RIL.

## Discussion

Seed germination is shortly followed by the mobilization of seed reserves from the seed storage organs or endosperm, providing essential energy to fuel growth until the seedling becomes photoautotrophic [15]. Because of its importance in seed germination and post-germinative growth, the mobilization of reserves such as starch, protein and lipids during germination has been extensively studied [15], [42]–[44]. In this study, we focused on the three traits (SDW, WMSR and SRUE) related to seed reserve utilization. The seedling establishment of the most RILs begins at day 6 of germination with the appearance of the radicle and shoot and the seed's energy reserves have mostly been exhausted at day 14 of germination. Therefore, the traits of seed reserve utilization were conducted at three germination stages (Day 6, 10 and 14). Those traits were identified in the dark to avoid the photosynthesis for energy usage in seed germination.

During seed germination, the dry weight of the growing seedling is always lower than that of the mobilized reserves due to respiration [16]. Similar results were observed in our results. Therefore, except for the WMSR, the SRUE, as the conversion efficiency of the mobilized reserve into seedling tissue, might be an important factor influencing vigorous seedling growth. In this study, we found that there were significant cultivar differences for seed reserve utilization that are in agreement with the findings of Soltani et al. [45] in wheat. The *japonica* variety Jiucaiqing had the higher WMSR, while the *indica* variety IR26 had the higher SRUE in the middle and late germination stages. Among the RIL population, the genetic difference in the SDW, WMSR and SRUE were also observed. These results indicate that genetic variation can be used in rice breeding programs for the improvement of seed reserve utilization.

In this study, we found that large seeds have the advantage of producing more vigorous seedlings, similar to previous results [16], [46]–[47]. The ISDW was significantly corrected with the SDW and WMSR, while no significant relationship between ISDW and SRUE. Due to the SDW affected not only the WMSR but also the SRUE in germinating seed, the SDW of small seed with higher SREU might be similar that of larger seed with low SRUE. Therefore, the lack of a significant positive relation between seed weight and seedling dry weight has also been reported in several studies [48]–[50].

Improving the trait of rice seed reserve utilization using conventional strategies is extremely difficult because it is a quantitative trait affected by both genetic and environmental factors. It is therefore necessary to perform QTL analysis of seed reserve utilization for marker assisted selection. Unconditional QTL mapping can explain cumulative gene actions from the beginning to the end of a specific process, and conditional mapping can identify the QTLs that act during a specific growth period but are not affected by the genes expressed in the previous stages [36]. In this study, a total of 20 unconditional QTLs were identified. Among them, 6, 6 and 11 unconditional QTLs were identified at day 6, 10 and 14 of germination stages, respectively. Similarly, a total of 21 conditional QTLs were identified. Among them, 6, 6 and 9 conditional QTLs were identified during day 0 to 6, 6 to 10 and 10 to 14 germination stages, respectively. These results showed that more QTLs were expressed during the late stage than during the early and middle stages. Combined using the conditional and unconditional QTLs, we can improve the seed reserve utilization traits not only during a specific germination period but also at the end of a specific germination stage by MAS.

We found that the additive QTLs were rarely co-localized among the three germination stages. Only the *qSDW1.2* was expressed at both day 10 and 14 germination stages, the *qWMSR4* was expressed at both day 6 and 14 germination stages, and the *qSRUE12.2* was expressed at both day 6 and 10 germination stages. These co-located QTLs might play important roles in controlling the seed reserve utilization during different germination stages. For the three traits of seed reserve utilization, the identification of co-localized additive QTLs only occurred in the genomic region RM6950–RM5759 of chromosome 1 for *qSDW1.2* and *qWMSR1*, the genomic region RM240–RM5404 of chromosome 2 for *qWMSR2* and *qSRUE2.2* and the genomic region RM519–RM7376 of chromosome 12 for *qSDW12* and *qWMSR12*. These co-localized QTLs could be very useful in the simultaneous improvement of more than one trait, as the desirable alleles of these additive QTLs (except for *qSRUE2.2*) were contributed by a single parent, IR26. Additionally, the joint analysis of the multi-development phenotypic values suggested that epistatic QTLs and QTL × development interactions were important components for seed reserve utilization, even though the degree of interaction was low.

**Figure 4:**
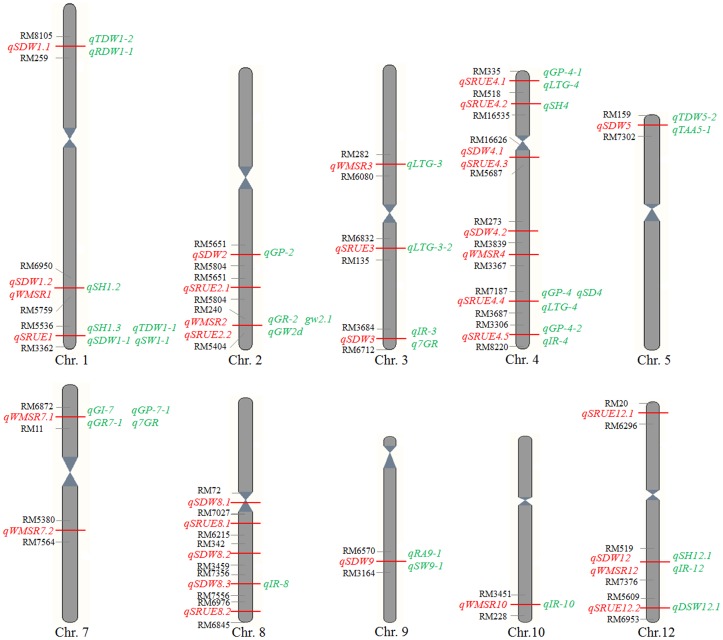
The co-located QTLs in this study with previously mapped QTLs in rice. The mapped QTLs in this study are shown on the left (red) and previously mapped QTLs are shown on the right (green) [8], [11]–[13], [19]–[25], [51]–[53].

By comparing the chromosomal positions of these additive QTLs, 36 QTLs identified in previous studies were found to map near the additive QTLs identified in this study (Fig. 4). The *qSDW1.2* and *qWMSR1* located to a position that coincided with the region of *qSH1.2* for shoot height [20], and the *qSDW1.1* coincided with the region of *qTDW1-2* for total seedling dry weight and *qRDW1-1* for root dry weight [8]. The *qSDW2* was on the similar location of *qGP-2* for germination percentage [12], and the *qSDW3* was similar with the location of *qIR-3* for imbibition rate [12] and *q7GR* for germination rate [24]. The *qSDW5* mapped near the region of *qTDW5-2* for total dry weight and *qTAA5-1* for total amylase activity [8], and the *qSDW8.3* was on the similar location of *qIR-8* for imbibition rate [12]. The *qSDW9* identified here was near the region of *qRA9-1* for root activity and *qSW9-1* for seed weight [8], and the *qSDW12* and *qWMSR12* was near the region of *qSH12.1* for shoot height [20] and *qIR-12* for imbibition rate [12].

Similarly, the *qWMSR2* and *qSRUE2.2* was on the similar location of *qGR-2* for germination rate [11], *gw2.1* for 1000-seed weight [51] and *qGW2d* for grain width [52]. The *qWMSR3* located to a position that coincided with the region of *qLTG-3* for low temperature germinability [25]. The *qWMSR7.1* was on the similar location of *qGR7-1* [8] and *q7GR* [24] for germination rate, *qGI-7* for germination index [11] and *qGP-7-1* for germination percentage [12]. The *qWMSR10* mapped near the region of *qIR-10* for imbibition rate [12].

Additionally, the *qSRUE1* identified here was near the region of *qTDW1-1* for total dry weight, *qSDW1-1* for shoot dry weight, *qSW1-1* for seed weight [8] and *qSH1.3* for shoot height [20]. The *qSRUE3* was near the region of *qLTG-3-2* for low temperature germinability [22]. The *qSRUE4.1* was on the similar location of *qGP-4-1* for germination rate [12] and *qLTG-4* for low temperature germinability [22], and the *qSRUE4.2* was similar with the region of *qSH4* for shoot height [20]. The *qSRUE4.4* located on the same region of *qSD4* for seed dormancy [53], *qLTG-4* for low temperature germinability [25] and *qGP-4* for germination percentage [11]. The *qSRUE4.5* was on the similar location of *qGP-4-2* for germination percentage and *qIR-4* for imbibition rate [13]. The *qSRUE12.2* mapped near the region of *qDSW12.1* for seedling dry weight [12]. However, there were no QTLs previously reported to be close to *qSDW4.1*, *qSDW4.2*, *qSDW8.1*, *qSDW8.2*, *qWMSR4*, *qWMSR7.2*, *qSRUE2.1*, *qSRUE4.3*, *qSRUE8.1*, *qSRUE8.2*, and *qSRUE12.1*, which indicates that these additive QTLs might be novel (Fig. 4).

Molecular evidence indicates that genes related to reserve mobilization and endosperm weakening could possibly affect seed germination [54]–[55]. Recently, several major QTLs for seed germination have been identified. For example, Wang et al. [11] identified one major QTL (*qGI*-*11*) for seed germination speed. Wang et al. [12] identified one major QTL (*qGR-2*) for seed germination under salt stress conditions. Recently, examination of the mechanism of seed vigor in rice has made significant progress. Fujino et al. [22] mapped a major rice QTL (*qLTG3-1*) for low-temperature germinability on chromosome 3. Fujino et al. [23] placed *qLTG3-1* in a 4.8-kb region of chromosome 3 using high-resolution mapping and revealed that the expression of *qLTG3-1* was tightly associated with the vacuolation of the tissues covering the embryo. This vacuolation may cause tissue weakening, resulting in the reduction of the mechanical resistance to the growth potential of the coleoptile. Seed reserve utilization is a very complex physiological process during seed germination. In this study, two major QTLs *qWMSR7.1* and *qSRUE4.3* were identified, and each QTL could explain more than 20% of the total phenotypic variance. Fine mapping the two QTLs are now in progress to elucidate the molecular mechanism of seed reserve utilization using near isogenic lines (NILs). Furthermore, the linked marker with the two major QTLs might be useful in marker-assisted selection to increase the seed reserve utilization in rice.

In a hypothetical cross of two cultivars, the trait values of produced RILs can be predicted by the effects of all the detected loci. The best RIL with maximum value would represent the best cross [41]. In this study, the best four cross combinations of each trait for the development of RIL populations were selected. It was found that some RILs present repeatedly in the novel parental combinations: for example, RIL 1750 in SDW and WMSR and RIL 1890 in WMSR and SRUE, indicating high general combining ability. To improve multiple traits, all the elite alleles might be pyramided into one cultivar as far as possible [41]. In this study, a total of 33 elite alleles could be pyramided by the combination of four RILs to improve the seed reserve utilization traits. These results showed that the selected RILs as new materials will be valuable in future rice breeding programs. The identified QTLs will be applicable to improve rice seed reserve utilization by MAS.
